# Multiple Metabolic Engineering Strategies to Improve Shikimate Titer in *Escherichia coli*

**DOI:** 10.3390/metabo13060747

**Published:** 2023-06-12

**Authors:** Taidong Bo, Chen Wu, Zeting Wang, Hao Jiang, Feiao Wang, Ning Chen, Yanjun Li

**Affiliations:** 1 College of Biotechnology, Tianjin University of Science and Technology, Tianjin 300457, China; 2 Key Laboratory of Industrial Fermentation Microbiology, Ministry of Education, Tianjin University of Science and Technology, Tianjin 300457, China; 3 National and Local United Engineering Lab of Metabolic Control Fermentation Technology, Tianjin University of Science and Technology, Tianjin 300457, China

**Keywords:** *Escherichia coli*, metabolic engineering, shikimate, fusion protein, shikimate kinase, quorum sensing

## Abstract

Shikimate is a valuable chiral precursor for synthesizing oseltamivir (Tamiflu^®^) and other chemicals. High production of shikimate via microbial fermentation has attracted increasing attention to overcome the unstable and expensive supply of shikimate extracted from plant resources. The current cost of microbial production of shikimate via engineered strains is still unsatisfactory, and thus more metabolic strategies need to be investigated to further increase the production efficiency. In this study, we first constructed a shikimate *E. coli* producer through the application of the non-phosphoenolpyruvate: carbohydrate phosphotransferase system (non-PTS) glucose uptake pathway, the attenuation of the shikimate degradation metabolism, and the introduction of a mutant of feedback-resistant 3-deoxy-D-arabino-heptulosonate 7-phosphate (DAHP) synthase. Inspired by the natural presence of bifunctional 3-dehydroquinate dehydratase (DHD)-shikimate dehydrogenase (SDH) enzyme in plants, we then designed an artificial fusion protein of DHD-SDH to decrease the accumulation of the byproduct 3-dehydroshikimate (DHS). Subsequently, a repressed shikimate kinase (SK) mutant was selected to promote shikimate accumulation without the supplementation of expensive aromatic substances. Furthermore, EsaR-based quorum sensing (QS) circuits were employed to regulate the metabolic flux distribution between cell growth and product synthesis. The final engineered strain dSA10 produced 60.31 g/L shikimate with a yield of 0.30 g/g glucose in a 5 L bioreactor.

## 1. Introduction

Shikimate (shikimic acid, SA) is a valuable compound, possessing six-carbon cyclitol with three stereogenic carbons and a carboxylic acid functional group [1]. It is widely used in the pharmaceutical field and is a precursor for the chemical synthesis of many anti-viral and anti-cancer drugs, such as the neuraminidase inhibitor oseltamivir phosphate (Tamiflu^®^) [2]. Shikimate is also a key chiral starting material to many chemical substances used in the chemical and cosmetic industries [3,4]. In 1885, shikimate was first isolated from *Illicium anisatum* (Japanese star anise) by Eijkman and was named “shikimic”, and its structure was elucidated several decades later [5]. Extraction from plant sources is currently a primary approach for shikimate production, which is still an expensive manufacturing process with the disadvantages of limited raw materials, low yield, complicated extraction procedure and environmental pollution [5,6]. Chemical synthesis of SA based on the Diels–Alder reaction has been successfully performed, which lacks commercial viability because of the production of hazardous waste, low yield, low enantiomeric purity, complicated synthetic steps and high cost [2]. With an increase in research on the shikimate pathway, microbial fermentation of shikimate has become an alternative potential and sustainable industrial production method [7,8]. Although different high-yield strains of shikimate have been successfully constructed, most of the titer, yield, or productivity remains far below the industrial requirements [9]. On the other hand, it is difficult to optimize the cultivation parameters, such as pH and temperature, for the cell biomass and product accumulation. Although shikimate is a primary metabolite, its production in engineered strains exhibited typical secondary metabolite patterns [10]. Substrate engineering and optimization is imperative for the high-level biosynthesis of shikimate [11]. Furthermore, the studies of process scale-up and product separation for shikimate production are currently scarce. Thus, lots of efforts concerning the shikimate production processes have to be emphasized for its industrial manufacture.

**Figure 1:**
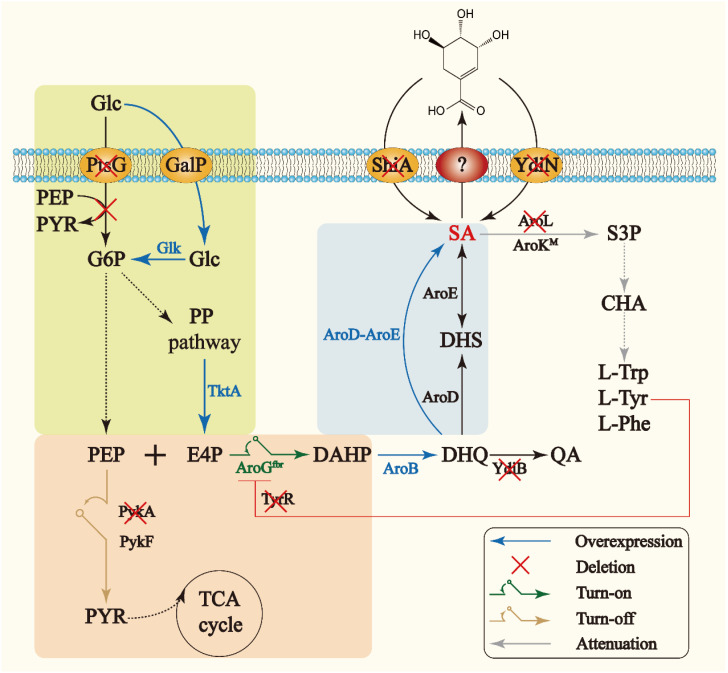
The biosynthetic pathway of shikimate in *E. coli* and the metabolic engineering strategies used in this study. PtsG, glucose PTS system EIICB; GalP, D-galactose transporter; ShiA and YdiN, putative shikimate transporters; Glc, glucose; PEP, phosphoenolpyruvate; PYR, pyruvate; G6P, glucose 6-phosphate; E4P, erythrose-4-phosphate; DAHP, 3-deoxy-D-arabino-heptulosonate 7-phosphate; DHQ, 3-dehydroquinate; QA, quinate; DHS, 3-dehydroshikimate; SA, shikimate; CHA, chorismate; L-Trp, L-tryptophan; L-Tyr, L-tyrosine; L-Phe, L-phenylalanine; *fbr*, feedback-resistant; PP pathway, pentose phosphate pathway; S3P, shikimate 3-phosphate; Glk, glucokinase; TktA, transketolase; AroG, 3-deoxy-7-phosphoheptulonate synthase; AroB, 3-dehydroquinate synthase; YdiB, quinate dehydrogenase; AroD, 3-dehydroquinate dehydratase; AroE, shikimate dehydrogenase; AroL/AroK, shikimate kinase I/II; M, mutation; PykA/PykF, pyruvate kinase. The solid line represents a one-step reaction and the dashed line represents multi-step reactions.

To date, different host strains including *Corynebacterium glutamicum* [6] and *Escherichia coli* (*E. coli*) [8,10] have been genetically engineered for the production of shikimate. *E. coli* is an attractive host because of its unambiguous genetic background, sophisticated genetic manipulation, rapid cell growth, and low nutrient acquirement [12,13]. In *E. coli*, the biosynthesis of shikimate initiates with the condensation of phosphoenolpyruvate (PEP) and erythrose 4-phosphate (E4P) to form 2-Dehydro-3-deoxy-D-arabino-heptonate 7-phosphate (DAHP), catalyzed by DAHP synthases (encoded by *aroG*, *aroF* and *aroH*). Subsequently, DAHP is sequentially converted to 3-dehydroquinate (DHQ), 3-dehydroshikimate (DHS), and shikimate, through the activities of DHQ synthase (encoded by *aroB*), 3-dehydroquinate dehydratase (DHD, encoded by *aroD*), and shikimate dehydrogenase (SDH, encoded by *aroE*), respectively. Shikimate is then transformed further to shikimate-3-phosphate by shikimate kinase (SK) I and II encoded by *aroK* and *aroL*, respectively (Figure 1). There are many challenges associated with the engineering of shikimate-overproducing *E. coli* strains, including product inhibition, insufficient precursor supply, byproduct formation, and competition between carbon flux and cell growth. To address these problems, several metabolic strategies have been well established in *E. coli* for achieving high production of shikimate over the past years. For example, feedback-resistant DAHP synthases were utilized to channel more metabolic flux to the shikimate pathway, the non-phosphoenolpyruvate: carbohydrate phosphotransferase system (non-PTS) glucose assimilation pathway was introduced to reinforce the supply of PEP for shikimate production, the quinate dehydrogenase was deleted to reduce the accumulation of quinate, and two putative shikimate transporters, ShiA and YdiN, were inactivated to prevent the shikimate reuptake [8,10,14]. However, other metabolic engineering strategies undoubtedly need to be investigated to further increase the technical parameters and reduce the cost of shikimate production, and meet the requirements of its industrial production. To block the downstream shikimate metabolism, the SKs are often inactivated, which makes the addition of expensive growth-dependent compounds compulsory. Other strategies regulating the expression of SK are thus needed to avoid this scenario. Strategies for reducing the commonly accumulated byproduct DHS, and for maintaining the carbon flux balance between microbial cell growth and product production are required as well.

In the present study, we constructed a shikimate high producer from scratch using the wild-type *E. coli* W3110 as a starting strain. The enhanced supply of precursors PEP and E4P was achieved by modifying the glucose uptake system and strengthening the expression of *tktA*, respectively. Subsequently, an artificial fusion protein of DHD-SDH was designed and applied to reduce DHS accumulation. Afterwards, an appropriate SK repressed mutant was selected to promote shikimate production without the supplementation of aromatic substances. Finally, the carbon source distribution in cell growth and shikimate production was regulated through a dual regulatory quorum sensing (QS) circuits. The obtained strain dSA10 produced 60.31 g/L shikimate with a yield of 0.30 g/g glucose after 64 h of fermentation in a 5 L bioreactor.

## 2. Materials and Methods

### 2.1. Strains and Reagents

The strains and plasmids used in this study are listed in Supplementary Table S1. *E. coli* DH5α was used as the cloning host and *E. coli* W3110 was used as the starting strain for genomic manipulations. *E. coli* strains were cultivated in LB medium at the required temperature. Ampicillin (100 μg/mL) and spectinomycin (50 μg/mL) were added if necessary, and IPTG (0.2 mM/L) and L-arabinose (2 g/L) were used as inducers. Primer STAR HS DNA polymerase was purchased from Takara Bio Co., Ltd. (Dalian, China). 2 × Rapid Taq Mix and ClonExpress ^®^ II One Step Cloning Kit were obtained from Vazyme Biotech Co., Ltd. (Ningjing, China). Genes and oligo-nucleotides were synthesized via GENEWIZ, Inc. (Suzhou, China).

### 2.2. Plasmid Construction

All primers used in this study are listed in Table S2. The gRNA targeting sequences are listed in Table S3. The plasmids pREDCas9 and pGRB used for CRISPR/Cas9-mediated gene editing system were kindly provided by Prof. Tao Chen in Tianjin University [15]. To generate a gRNA-expressing plasmid for gene editing, the gRNA-coding sequence was obtained by annealing a pair of reverse complementary single-stranded oligonucleotides, and then assembled into reverse PCR-linearized pGRB through homologous recombination using ClonExpress^®^ II. To generate the pSTV28-*mCherry*-*egfp* plasmid, pSTV28 was linearized using *Eco*R I and *Hin*d III, the P_easR-C_ promoter, *mCherry* gene, P_easS_ promoter and *egfp* gene were ligated together via overlap PCR, and then assembled with the linearized plasmid.

### 2.3. CRISPR/Cas9-Mediated Genome Editing

CRISPR/Cas9-mediated genome editing method [15] is used to integrate or delete genes. For recombination, the plasmid pREDCas9 was transformed into *E. coli* competent cells using a CaCl_2_-mediated approach. To transform gRNA plasmids and DNA fragments for recombination into cells, *E. coli* competent cells harboring plasmid pREDCas9 were cultured in LB medium with 50 μg/mL spectinomycin at 32 °C. When the OD_600_ reached 0.1–0.2, 0.1 mM isopropyl β-D-1-thiogalactopyranoside (IPTG) was added to induce the expression of λ red recombinases. When the OD_600_ reached 0.6–0.7, cells were harvested, and then gRNA plasmids (100 ng) and DNA fragments (200 ng) were co-transformed into the competent cells using an Eppendorf Eporator (Taufkirchen, Germany) at 1.85 kV. After electroporation, the transformants were recovered in 1 mL LB medium at 32 °C for 2 h before plating on LB medium with 50 μg/mL ampicillin and 50 μg/mL spectinomycin for confirmation of the presence of gRNA and pREDCas9 plasmids, respectively. After overnight culturing at 32 °C, randomly selected colonies were screened via colony PCR and the positive transformants were further confirmed via DNA sequencing. When engineering was completed, the selected colonies were cultured in LB medium with 0.2% L-arabinose at 32 °C to induce the expression of gRNA targeting on pGRB plasmid for plasmid elimination. The plasmid pREDCas9 can be eliminated by culturing at 42 °C.

### 2.4. Shake Flask Fermentation

The engineered bacteria cultured on agar slants were transferred to a 500 mL covered baffled shake flask containing 30 mL of seed medium and incubated at a shaker temperature of 37 °C and a speed of 200 rpm for 10 h. The seed cultures contained (per liter) 20 g glucose, 4 g yeast extract, 1.6 g citric acid, 1.2 g (NH_4_)_2_SO_4_, 5.6 g K_2_HPO_4_·3H_2_O, 1.6 g MgSO_4_·7H_2_O, 0.5 g choline chloride, 2.8 mg FeSO_4_·7H_2_O, 1.2 mg MnSO_4_·H_2_O, 1.3 mg VB_1_, 1.3 mg VB_3_, 1.3 mg VB_5_, 2 mg VB_7_ (biotin), 1.3 mg VB_12_ and 2 mL trace element solution, pH 7.0–7.2. The trace element solution contained (per liter) 10 g CaCl_2_·2H_2_O, 0.6 g CuSO_4_·2H_2_O, 4.9 g CoCl_2_·6H_2_O, and 6.4 g ZnSO_4_·2H_2_O. The seed cultures (3 mL) were inoculated in 500 mL shake flasks containing 30 mL of fermentation medium and incubated at 37 °C for 24 h or 72 h with a shaking speed of 200 rpm. The fermentation medium contained (per liter) 10 g glucose, 2 g yeast extract, 2 g citric acid, 1.6 g (NH_4_)_2_SO_4_, 8.5 g K_2_HPO_4_·3H_2_O, 2.5 g MgSO_4_·7H_2_O, 0.5 g choline chloride, 75 mg FeSO_4_·7H_2_O, 0.15 mg VB_1_, 0.15 mg VB_3_, 0.15 mg VB_5_, 0.15 mg VB_12_, 0.1 mg VB_7_ and 2 mL trace element solution, pH 7.0–7.2.

### 2.5. Fed-Batch Fermentation in a 5 L Fermenter

The strains were pre-cultured on agar slant at 37 °C for 12 h. The agar slant cultured cells were then transferred into a 5 L fermenter containing 3 L seed medium. The seed and fermentation media in the fermenter were the same as those used in the shake flasks. When the OD_600_ reached 12–15, 450 mL of culture broth was retained for the fed-batch fermentation and fresh fermentation medium was added immediately to make a final volume of fermentation broth of 3 L. The pH was automatically controlled throughout the fermentation process by adding ammonium hydroxide (25%, *v*/*v*) at 37 °C, and the dissolved oxygen was maintained above 25% by varying the stirring rate and aeration. When the substrate glucose was depleted, sterile glucose solution (80%, *w*/*v*) was supplemented in appropriate amounts, and the glucose concentration was maintained below 2 g/L.

### 2.6. Analytical Methods

Cell growth (OD_600_) was monitored via a UV spectrophotometer at 600 nm. The glucose concentration was determined using SBA-40C biosensor. The shikimate content in the fermentation broth was detected via HPLC (LC-20AT; Shimadzu, Kyoto, Japan) equipped with an Aminex HPX-87H column (Bio-Rad, Hercules, CA, USA); the mobile phase was 5 mM/L sulfuric acid, the flow rate was 0.5 mL/min, the column temperature was 30 °C, and the detection wavelength was 215 nm.

The QS system with bifunctional switch was evaluated by determining the fluorescences of eGFP and mCherry. In detail, the stains dSA09-1, dSA09-2 and dSA09-3 grew overnight at 220 rpm in 30 mL of LB medium with Cm^R^ at 37 °C. Using the overnight culture, a new flask of 50 mL LB was inoculated to a diluted cell culture of 1:100 supplemented with antibiotics, and samples were collected for analysis every 2 h. The 200 μL sample was centrifuged at 12,000 rpm and the precipitate was then resuspended in 1 mL 0.9% NaCl solution. This process was repeated and 200 μL suspension was taken for fluorescence detection. The assessment was carried out using an infinite M200PRO microplate reader (TECAN). The excitation and emission wavelengths for green fluorescence were at 475 ± 10 nm and 520 ± 10 nm, and for red fluorescence were at 565 ± 10 nm and 630 ± 10 nm, respectively.

### 2.7. Statistical Analysis

Data represent the mean and standard deviation (SD) of three independent experiments. One-way analysis of variance (ANOVA) and Dunnett’s multiple comparison test were used to determine significant differences between the data. 0.01 < *p* < 0.05 was considered statistically different, 0.001 < *p* < 0.01 indicates significant, while *p* < 0.001 indicates highly significant.

## 3. Results and Discussion

### 3.1. Strategies for Construction of a Basal Shikimate Production Strain

#### 3.1.1. Modification of Glucose Uptake Pathway

Glucose uptake in *E. coli* is mediated by the PEP consuming the PTS pathway. In order to increase the availability of PEP for shikimate synthesis, it has been a common strategy to inactivate and replace the PTS with a PEP-independent, but an ATP-dependent uptake and phosphorylation system consisting of the glucose facilitator and the glucokinase [8,9,16,17]. This approach is also used to produce shikimate pathway extended compounds including aromatic amino acids and zosteric acid [18,19]. After the inactivation of PTS system, the strain’s ability to assimilate glucose is greatly affected. In order to improve glucose uptake, the *galP* gene encoding galactose permease in *E. coli* [9,18] or the *glf* gene in *Zymomonas mobilis* [20] are usually introduced. In addition, the non-PTS inositol permease IolT1 [21] has been widely used in *C. glutamicum* as an alternative glucose uptake route for the production of a variety of chemicals. In this study, we compared the effects of the introduction of *galP* from *E. coli* (resulting in strain dSA01-1) and *iolT1* (*Cgl0181*) from *C. glutamicum* (resulting in strain dSA01-2), at the locus of *ptsG* gene in *lacI*-deficient *E. coli* W3110 strain (dSA00), on the cell growth. The expression of *galP* or *iolT1* was driven by the promoter of the *ptsG* gene, and thus was subjected to similar transcriptional regulations as *ptsG*. Meanwhile, the combined expression of the glucokinase encoding gene *glk* from *E. coli* (strain dSA02-1) or *glk1* (*cg2399*) from *C. glutamicum* (strain dSA02-2) were also conducted.

**Figure 2:**
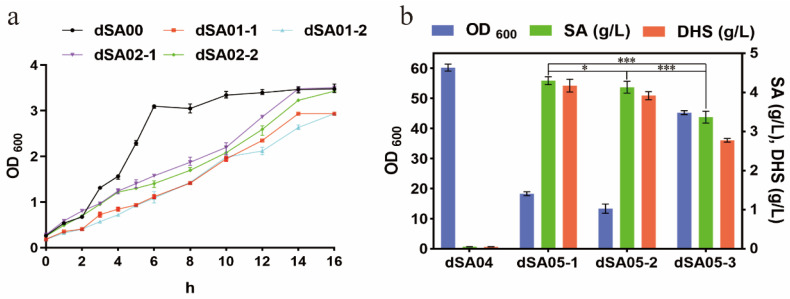
Modification of glucose uptake pathway and introduction of feedback-resistant DAHP synthase. (**a**) Effects of application of non-PTS systems from *E. coli* and *C. glutamicum* on the bacterial growth. (**b**) Effects of expression of *aroG*^fbr^ mutants on biomass accumulation, shikimate and DHS production. Values are presented as mean ± SD. * *p* < 0.05, indicates statistically different; *** *p* < 0.001, indicates highly significant.

These strains were cultivated in M9 minimum medium and the growth curves were plotted (Figure 2a). Compared with that of the control strain dSA00, the growth of *ptsG*-inactivated strains dSA01-1 and dSA01-2 was distinctly decreased. Further expression of glucokinases in strains dSA02-1 and dSA02-2 slightly promoted the bacterial growth, which, however, was not fully restored compared to that of the dSA00. These observations were inconsistent with those previously reported [18,22], which might be due to the different expression strength of these genes since the plasmid was not utilized in this study. The combination of endogenous genes showed better effects than that of *C. glutamicum*-derived genes, and thus the strain dSA02-1 was used for further experiments. E4P is another precursor of the shikimate pathway, and the *tktA* gene was overexpressed to enhance the availability of E4P, resulting in strain dSA03.

#### 3.1.2. Introduction of Feedback-Resistant Mutant aroG^fbr^

At first, we weakened the catabolic pathway of shikimate and inactivated the pathways contributing to quinate synthesis and shikimate assimilation. In *E. coli*, SK1 (encoded by *aroK*) and SK2 (encoded by *aroL*) are responsible for converting shikimate to shikimate-3-phosphate, and SK2 plays a dominant role in the shikimate pathway [23]. In order to facilitate shikimate accumulation, disruption of the two genes seems to be a prerequisite for constructing shikimate producers [8,10,16,17,24]. However, the complete elimination of SKs would block the synthesis of chorismate and its downstream derivatives, such as aromatic amino acids [17]. Herein, we attempted to explore alternative strategies and only inactivate *aroL* in dSA03 by inserting the *aroB* gene, encoding DHQ synthase, under the P_trc_ promoter. Quinate and DHS are commonly observed as the major byproducts for shikimate production [8,24]. Quinate accumulation is mainly attributed to quinate dehydrogenase (encoded by *ydiB*), which catalyzes the conversion of DHQ into quinate [8,25,26]. Therefore, the *ydiB* gene was deleted to diminish quinate accumulation. Afterwards, the shikimate transporter ShiA [27] and the putative transporter YdiN [10] were knocked out, thereby inhibiting the re-uptake of shikimate in the medium. The resulting strain was designated dSA04.

In shikimate pathway, the carbon flow to chorismate is primarily controlled at the first reaction of DAHP synthase. In *E. coli*, three genes, *aroG*, *aroF*, and *aroH*, encode the DAHP synthase isozymes that are subject to feedback inhibition by L-phenylalanine, L-tyrosine, and L-tryptophan, respectively [28]. In wild-type *E. coli* grown in minimal medium, about 80, 20, and 1% of the total DAHP synthase activities are contributed by the *aroG*, *aroF*, and *aroH* products, respectively [29]. Although the metabolic fluxes to the two precursors PEP and E4P were reinforced and the so-called rate-limiting enzyme DHQ synthase [30] was overexpressed, the strain dSA04 could hardly accumulate shikimate (Figure 2b), indicating that the feedback-resistant DAHP synthase mutant needed to be introduced.

We chose three mutants of *aroG* that encodes DAHP synthase isozyme with the highest activity, *aroG*^D146N^ [19,31], *aroG*^S180F^ [32] and *aroG*^S211F^ [33], to express under P_trc_ promoter, resulting in strains dSA05-1, dSA05-2 and dSA05-3, respectively. The shake flask fermentation showed that all these strains produced considerable amounts of shikimate, while accumulated biomass decreased, which were measured after 24 h of fermentation (Figure 2b). The shikimate titer of dSA05-1 and dSA05-2 reached 4.30 g/L and 4.13 g/L, respectively, significantly higher than that of dSA05-3 (3.36 g/L); however, the growth of these two strains were severely impaired. The dSA05-3 showed less decreased biomass, 25.2% lower than that of the control, which was suitable for further modifications. These results suggested that it was crucial to select an appropriate *aroG* mutant, as different mutations of this gene could affect the activity of DAHP synthase to different extents [34]. In the PTS^-^ strains, pyruvate, one of the most important central metabolites, is generated from PEP only by pyruvate kinases. Overexpression of *aroG* would drain more PEP to the shikimate pathway [32] and thus diminish the pyruvate supply for other cellular metabolisms, particularly the tricarboxylic acid (TCA) cycle, which in turn, negatively affected the bacterial growth. Additionally, almost equal amounts of DHS as shikimate were accumulated in the cultures of these strains (Figure 2b).

### 3.2. Strategy for Enhancing Shikimate Production and Reducing DHS Accumulation

**Figure 3:**
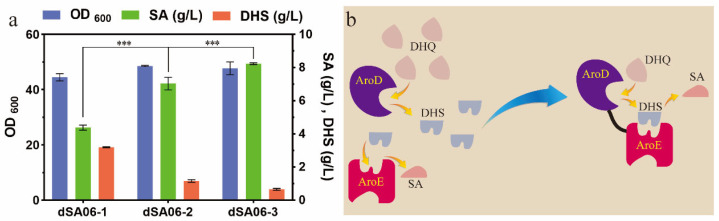
Optimization of *aroD* and *aroE* gene expression patterns. (**a**) Effects of expression of *aroD* and *aroE* separately or *aroD*-*aroE* in combination on biomass accumulation, shikimate and DHS production. (**b**) The proposed mechanism for enhanced conversion of DHS to shikimate by fused DHD-SDH, increasing the effective concentration of DHS through the proximity of the two domains. Values are presented as mean ± SD. *** *p* < 0.001, indicates statistically highly significant.

We enhanced the expression of *aroD* in dSA05-3, resulting in the strain dSA06-1. In shake flask fermentations (Figure 3a), the shikimate titer was increased by 29.9% to 4.37 g/L, whereas the production of DHS was also increased by 15.2%, compared with those of dSA05-3. Subsequently, *aroE* was overexpressed in dSA06-1. The resultant strain dSA06-2 produced significantly more shikimate (7.03 g/L) and less DHS (1.16 g/L), in comparison with dSA05-3 and dSA06-1. These results indicated that overexpression of SDH was indeed beneficial for driving more DHS to shikimate and thus increased the production of shikimate. However, a considerable quantity of DHS still remained in the cultures. Researchers [8,35] observed that the accumulation of DHS was mainly caused by hydroaromatic equilibration; the redox nature of AroE enables the reverse reduction in shikimate to DHS. On this account, it seems inappropriate to further promote the expression of *aroE*. Although the dual specificity quinate/shikimate dehydrogenase YdiB has been designated as quinate dehydrogenase, some mutants were reported to have increased activity toward shikimate [36]. We overexpressed the mutant gene *ydiB*^S67A^ in dSA06-2, but productions of shikimate and DHS of the resultant strain remained unchanged.

Unlike in bacteria, such as *E. coli*, *aroD* and *aroE* in plants are fused to form a single gene encoding the bifunctional DHD-SDH enzyme. The crystal structure of the *Arabidopsis thaliana* enzyme shows that the active sites of DHD and SDH are localized in close proximity and face each other, which facilitates an optimal, local DHS concentration for effective SDH catalysis (Figure 3b) [37]. Inspired by this natural existence, we constructed an artificial gene by fusing *E. coli aroD* and *aroE* with the linker from *A. thaliana*, and introduced it into the genome of dSA05-3. The resultant strain dSA06-3 produced 8.23 g/L shikimate and only 0.66 g/L DHS after 24 h of fermentation, which was decreased by 76.3% compared with that of dSA05-3. Very recently, the mutant AroE^L241I/T61W^ with reduced affinity for shikimate and increased or unchanged affinity for DHS was utilized; the DHS titer was reduced by 52.1% and the SA titer was increased by 12.9%, reaching 88.50 g/L [8]. In this study, we exploited an alternative strategy to reduce the DHS accumulation by mimicking the natural bifunctional enzyme from plants.

### 3.3. Strategy for Attenuation of aroK Expression

Despite the inactivation of SK II, the SK I expressed by the retained *aroK* gene prevented the strain from completely accumulating shikimate. In the majority of publications, *aroK* was also deleted to block shikimate metabolism [8,10,16,17,24,38,39]. In these cases, the engineered shikimate producers are auxotrophic. During fermentation, three aromatic amino acids and aromatic vitamins, such as p-hydroxybenzoate, p-aminobenzoate and 2,3-dihydroxybenzoate, must therefore be added to the medium to maintain normal host growth, thus increasing the cost of industrial production. To overcome this problem, different strategies have been developed to dynamically repress the expression of *aroK*, such as the employment of growth phase-dependent regulation [40], L-arabinose-induced tunable switch [9], and light-controlled switch [41]. The dynamic regulation of *aroK* expression to decouple biomass formation and shikimate production avoids the addition of expensive aromatic amino acids and other aromatic compounds, however, the installment of special devices or the supplementation of chemical inducers are needed. In this study, we attempted to use alternatively the static regulation strategy to achieve controllable expression of *aroK*. To date, crystal structures of several bacterial SKs (encoded by *aroK*) have been reported, including that from *E. coli* [42], *Mycobacterium tuberculosis* [43], *Acinetobacter baumannii* [44], and *Helicobacter pylori* [45,46]. For the *Hp*AroK, residues interacting with functional groups in shikimate can be considered to form three subsites on the protein: (i) C_X_, which contacts a carboxyl moiety of shikimate; (ii) O_CORE_, which interacts with two hydroxyl groups of shikimate; and (iii) O_LID_, which interacts with a trans hydroxyl group of shikimate (Table 1) [45,46].

**Figure 4:**
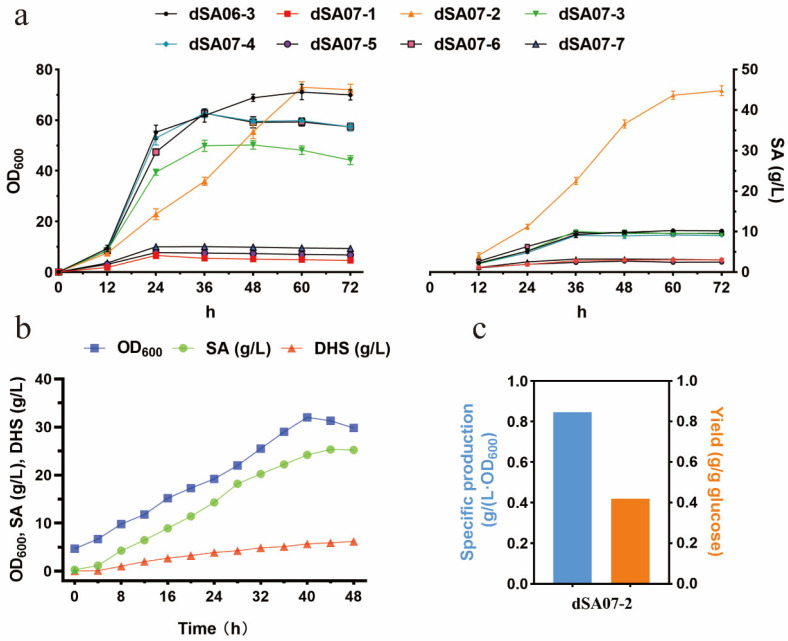
Effects of weakening *aroK* gene on shikimate fermentation. (**a**) Cell growth and shikimate production of strains harboring different *aroK* mutants in 500 mL shake flasks. (**b**) Fermentation parameters of dSA07-2 in a 5 L fermenter. (**c**) Specific production and yield of dSA07-2 in a 5 L fermenter. The values are derived from a representative batch culture selected from different replicates.

The site-directed mutagenesis of these residues was conducted and the enzyme activities of these mutants were characterized [45]. Herein, we constructed six mutants of *Ec*AroK in the genome of the strain dSA06-3, respectively, which correspond to the characterized *Hp*AroK mutants at the shikimate-binding residues (Table 1). Shake-flask fermentations of these strains were conducted using dSA06-3 and the *aroK*-deleted strain dSA07-1 as controls (Table 1). The growth of strains dSA07-5 (AroK^R60K^) and dSA07-7 (AroK^R140A^) were severely inhibited, in accordance with the strictly conserved property of these residues as revealed for *Hp*AroK (Figure 4a), and thus the shikimate titers were low. The strains dSA07-3 (AroK^V47I^), dSA07-4 (AroK^F51W^) and dSA07-6 (AroK^K118A^) presented slightly impaired growth compared with dSA06-3, indicating that these mutation residues are relatively flexible for maintaining SK activity and bacterial biomass; these four strains also produced comparable amounts of shikimate (Table 1). Interestingly, the growth of dSA07-2 (AroK^M13A^) was repressed initially, but was increased gradually till the value of dSA06-3; the shikimate titer of dSA07-2 reached 44.80 g/L, which was 3.4-fold higher than that of the titer of dSA06-3 (10.23 g/L). These results indicated that the utilization of depressed expression of AroK is a feasible strategy for high level production of shikimate and selection of an appropriate mutant is crucial; the corresponding mutant *Hp*AroK^M10A^ retains 38% that of the enzyme activity of the wild type (Table 1). The introduction of depressed mutants of a key enzyme guarantees the accumulation of the product while it also maintains a certain amount of downstream metabolic flux supporting the bacterial growth. This strategy of static regulation of key enzymes avoids the supplementation of expensive chemicals and the application of complicated fermentation processes. Similarly, the selection of leaky mutants is a common strategy in traditional breeding via mutagenesis. In metabolic engineering studies, the application of mutants with decreased activity has been frequently reported, for example, the *ilvA*^S97F^ encoding threonine dehydratase was introduced for accumulating threonine [47], and the *hom*^V56A^ was utilized to attenuate the threonine pathway branch for enhancing lysine production [48].

We carried out the fed-batch fermentation of the strain dSA07-2 in a 5 L bioreactor. As shown in Figure 4b,c, the cell growth was inhibited and therefore only 27.51 g/L shikimate was produced with a yield of 0.42 g/g glucose; the production of DHS reached 6.19 g/L. We speculated that the shikimate pathway might compete for more metabolic flux from glycolysis at the early stage of fermentation in the bioreactor system, adversely influencing the bacterial growth and shikimate production.

### 3.4. Strategy for Dynamic Regulation between the Shikimate Pathway and Central Metabolism

Current strategies of strain improvement are mainly focused on enhancing the biosynthetic pathway of the target product. However, excessive metabolic flux creates metabolic imbalances, which lead to growth retardation and ultimately limit the yield of the product. To control the metabolic flux distribution between cell growth and product synthesis, dynamic regulation strategies need to be exploited, among which, the QS system regulated by cell density, offers a promising dynamic control strategy that is pathway-independent and does not require exogenous inducers. The well-known QS modules employed in metabolic engineering mostly rely on the ability of LuxR and its homologues to activate gene expression upon binding diffusible QS signals, such as acyl homoserine lactone (AHL) [49,50,51]. Recently, an Esa QS system from *Pantoea stewartii* has been engineered to automatically down-regulate the competing pathway, significantly improving the production of myo-inositol, glucaric acid and shikimate [52]. Unlike most LuxR homologues, EsaR can act as both transcriptional activator and repressor, which has attracted increasing interest in the field of metabolic engineering [53,54,55,56,57,58].

**Figure 5:**
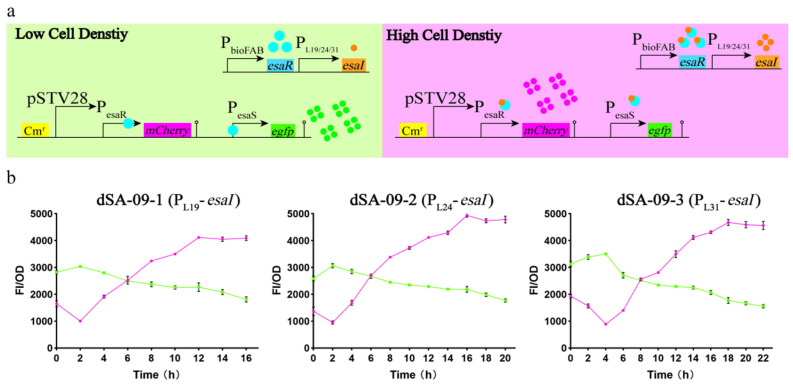
Overview of EsaR-based QS circuits. (**a**) Architecture of the P_esaR_ and P_esaS_ QS circuits. The P_bioFAB_-*esaR* and *esaI* (under the control of three promoters: P_L19_/P_L24_/P_L31_) expressing cassettes were inserted into the genome. The P_esaR_-*mCherry* and P_esaS_-*egfp* were expressed in pSTV28 vector and introduced into these strains. (**Left**) At low cell densities, EsaR binds to the promoters P_esaR_ and P_esaS_, and *mCherry* expression is off and *egfp* expression is on. (**Right**) At high cell densities, buildup of AHL produced by EsaI leads to the disruption of EsaR binding, turns on *mCherry* expression and turns off *egfp* expression. (**b**) Red and green fluorescence curves showing the response of P_esaR_ and P_esaS_ to varing *esaI* expression levels in these strains, respectively (dSA09-1: P_L19_-*esaI*; dSA09-2: P_L24_-*esaI*; dSA09-3: P_L31_-*esaI*).

In this study, we attempted to adopt the EsaR-based QS circuits to dynamically regulate the metabolic flux distribution between shikimate pathway and central metabolism. To this end, we first tuned the switching dynamics of these circuits using the expressions of *egfp* and *mCherry* as indicators. In our system, the *esaR*^170V^ gene was inserted into the genome under the control of a constitutive promoter apFAB104 (obtaining dSA08), and subsequently the *esaI* gene (encoding AHL synthase) was genome-integrated under the control of three different promoter and ribosome binding site (RBS) variants (denotated as L19, L24 and L31) (Table S4) [52], resulting in strains dSA09-1, dSA09-2 and dSA09-3, respectively. P_esaR_ is a natural EsaR-repressed promoter, whereas P_esaS_ is a natural EsaR-activated promoter. The cassettes of modified P_esaR-C_ promoter [59] driving *mCherry* expression and P_esaS_ promoter [52] driving *egfp* expression were inserted into the medium-copy plasmid pSTV28, which was then introduced into these strains. At low bacterial cell densities, EsaRI70V binds P_esaR-C_ promoter to repress *mCherry* transcription, and binds P_esaS_ promoter to activate *egfp* transcription. At high cell densities, accumulation of AHL occurs as it is produced by EsaI, which eventually leads to disruption of EsaRI70V binding, activating *mCherry* expression and deactivating *egfp* expression (Figure 5a). Shake flask cultivations of these strains harboring *mCherry or egfp* expression plasmid were conducted and red and green fluorescence were detected. The red fluorescence of these strains was indeed switched to the up-regulation mode over time, and the opposite was true for the green fluorescence (Figure 5b). The switching time for both red and green fluorescence of dSA09-1 and dSA09-2 was observed at 2 h, earlier than that of dSA09-3 (at 4 h). The same timing of P_esaS_ down-regulation and P_esaR-C_ up-regulation may have indicated that the two promoters had similar sensitivity to AHL [56]. The value of red fluorescence of dSA09-2 was the highest, whereas the green fluorescence of these strains showed approximately the same values. Therefore, we chose dSA09-2 with relatively high sensitivity and strong expression of red fluorescent protein for further study. In conclusion, by changing the AHL accumulation rate, which was controlled by varying the expression level of *esaI*, we tuned the up- and down-regulation modules.

**Figure 6:**
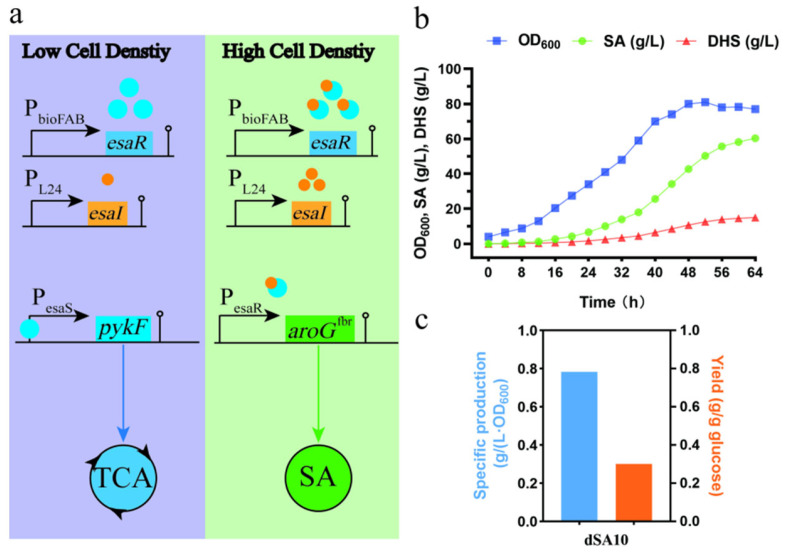
Application of the QS circuits for shikimate fermentation. (**a**) Schematic of the dual regulatory strategy. Increasing cell density triggers P_esaR_ and P_esaS_ switches, up-regulating *aroG^fbr^* expression and down-regulating *pykF* expression, respectively. (**b**) Fermentation parameters of the dSA10 strain with QS circuits in a 5 L fermenter. (**c**) Specific production and yield of dSA10 in a 5 L fermenter. The values are derived from a representative batch culture selected from different replicates.

As previously explained, the DAHP synthases control the first reaction of the shikimate pathway. The pyruvate kinases (encoded by *pykF* and *pykA*) catalyzed the reaction converting PEP to pyruvate, which is the entry substrate for the TCA cycle. The two reactions, at the point of shikimate pathway branching off from glycolysis, compete with PEP either for shikimate production or for cell growth. We attempted to apply the EsaR-based QS circuits at this branch point, enabling the bacterial cells switching from “growth mode” to “production mode” in a completely autonomous fashion. To do so, we deleted the *pykA* gene of the strain dSA09-2, and replaced the native promoters of *pykF* and *aroG*^fbr^ with P_esaS_ and P_esaR-C_ promoters, respectively (Figure 6a). The fed-batch fermentation of the resultant strain dSA10 was carried out in a 5 L bioreactor. The previously observed growth retardance of dSA07-2 was alleviated in dSA10. The shikimate titer achieved 60.31 g/L after 64 h of fermentation (Figure 6b), which was 138.2% higher than that of the strain dSA07-2 (Figure 4b). The accumulation of byproduct DHS reached 15.21 g/L. The specific production was 0.78 g/(L·OD_600_), and the yield was 0.30 g/g glucose (Figure 6c), which was 28.5% lower than that of dSA07-2. The reason for the decline of the yield might be that more carbon source was channeled into the TCA cycle before the transition from growth mode to production mode. Notably, the strain dSA10 was able to balance growth and production by driving more metabolic flux from the central carbon metabolism at a suitable switching time.

Microbial production of SA has gained great interests and many metabolic engineering studies constructing shikimate producers have been reported, some of which achieved high level production of shikimate (Table 2). However, further metabolic engineering strategies are needed to lower the production cost by microbial fermentation, making it more competitive compared to other production methods. The present study presents new strategies, which may contribute to guide the development of more efficient and robust shikimate producing strains. Especially, the combination and the order of introduction of genetic modifications revealed in this study is informative for constructing shikimate producers.

## 4. Conclusions

This study reported the development of a high-level shikimate producer starting from the wild-type *E. coli* W3110. Following the construction of a basal shikimate producing strain using metabolic strategies established in the litterateurs, we explored several alternative strategies to increase shikimate production. To reduce the accumulation of the byproduct DHS, researchers enhanced NADPH supply or introduced mutated SDH. We found that an artificial fusion protein of DHD-SDH was also helpful for this purpose, which might be due to the effective supply of DHS from DHD for SDH catalysis. In the published literatures, the SKs were often inactivated to prevent the metabolism of shikimate to downstream substances. Alternatively, we optimized a depressed SK mutant that drastically facilitated the accumulation of shikimate and avoided the necessity of adding expensive aromatic amino acids and vitamins. Additionally, the dual regulatory QS system was observed to be useful for alleviating retarded cell growth before transition to the production mode. These strategies may provide valuable references for further research on strain engineering to produce shikimate and related products.

## Figures and Tables

**Table 1:** Site-directed mutagenesis of shikimate-binding residues.

Engineered Strains	*Ec* AroK Mutants	Corresponding *Hp*AroK Mutants	Relative Activity of *Hp*AroK Mutants (%) [46]	Characteristics [46]
dSA06-3	WT	WT	100	-
dSA07-1	none (∆*aroK*)	-	-	-
dSA07-2	M13A	M10A	38	O_CORE_: contacts with a hydroxyl group of shikimate; relatively conserved
dSA07-3	V47I	V44I	N.A.	O_LID_: interacts with a *trans* hydroxyl group of shikimate; relatively conserved
dSA07-4	F51W	F48W	N.A.	O_LID_: interacts with a *trans* hydroxyl group of shikimate; relatively conserved
dSA07-5	R60K	R57K	2	C_X_: contacts with a carboxyl of shikimate; strictly conserved
dSA07-6	K118A	E114A	82	O_CORE_: contacts with a hydroxyl group of shikimate O_LID_: interacts with a *trans* hydroxyl group of shikimate; relatively conserved
dSA07-7	R140A	R132A	5	C_X_: contacts with a carboxyl of shikimate; strictly conserved

Shikimate binds to residues from three subsites: C_X_, O_CORE_ and O_LID_; WT: wild type; N.A.: not available.

**Table 2:** Production of shikimate using engineered *E. coli* strains.

*E. coli* Strains	Strategies	Shikimate Titer	Ref.
SP1.1pts/pSC6.090B	inactivation of *aroL*, *aroK*, *ptsH*, *ptsI*, *and crr*; overexpression of *aroB*, *glf*, *glk*, *aroF*^fbr^, *tktA*, *aroE*, and *serA*	87.00	[60]
PB12.SA22	inactivation of *aroK* and *aroL*; coexpression of *aroG*^fbr^, *aroB*, *tktA*, and *aroE*; inactivation of PTS	7.05	[16]
AR36	inactivation of PTS *aroK*, *aroL*, and *pykF*; overexpression of *aroB*, *tktA*, *aroG*^fbr^, *aroE*, *aroD*, and *zwf*	43.00	[17]
SA116	inactivation of *aroK* and ar*oL*; integration of *aroG*^fbr^, *aroB*, *aroE*, and *tktA*; overexpression of *csrB*, *pps,* and *pntAB*	3.12	[40]
P9	switch regulation of *aroK*; inactivation of *araC*, *pta*, *ptsG*, *aroL*, *trpR*, and *pykF*	13.15	[61]
SA5/pGBAE	inactivation of *ptsH*, *ptsI*, *crr, aroK* and *aroL*; integration of *glk*, *galP*, *aroG*, *aroB*, *tktA* and *aroE*; integration of *ppsA* at *tyrR*	27.41	[62]
DS7	PTS system was genomically replaced by a glucose facilitator protein gene, *Zmglf*, from *Z. mobilis*; inactivation of *aroK* and *aroL*; overexpression of *tktA*, *aroG*, *aroB*, and *aroE*	12.63	[63]
Inha 224	inactivation of *aroK*, *aroL*, *tyrR*, *ptsG*, *pykA*, and *shiA*; overexpression of *aroB*, *aroD*, *aroG*, *aroF*, *ppsA*, *aroE*, *galP*, and *tktA*	101.00	[10]
SA09	inactivation of *aroK* and *aroL*; overexpression of *glyA*, *talB*, *aroG*, *aroD*, and *aroE*^L241I/T61W^, *tktA*; deletion of *ptsH*, *ptsI*, *pykF*, *dhal*, and *ydiB*	126.40	[8]
dSA10	*ptsG* was genomically replaced by *galP*; inactivation of *aroL*, *tyrR*, *ydiB*, *shiA*, *ydiN* and *pykA*; mutation of *aroK*^M13A^; overexpression of *glk*, *tktA*, *aroB*, and *aroD*-*aroE*; dynamic regulation of *pykF* and *aroG*^S211F^	60.31	This study

## Data Availability

Data are available in the main article and the Supplementary Materials. The raw data are available from the corresponding author upon reasonable request.

## Supplementary Materials

The following supporting information can be downloaded at: https://www.mdpi.com/article/10.3390/metabo13060747/s1, Figure S1: The HPLC chromatograph of shikimate and DHS; Table S1: Strains and plasmids used in this study; Table S2. Primers used in this study; Table S3. The gRNA targeting sequences used in this study; Table S4. Gene and promoter sequences involved in the QS circuits.
