# Supplementary material for: Multiple Metabolic Engineering Strategies to Improve Shikimate Titer in Escherichia coli
Source: Metabolites. 2023 Jun 12;13(6):747. doi: 10.3390/metabo13060747 (PMC10305709; doi:10.3390/metabo13060747)
Supplement: Supplementary file 1 [file metabolites-13-00747-s001.zip › metabolites-2302336-supplementary.pdf]

Multiple Metabolic Engineering Strategies to Improve Shikimate Titer in *Escherichia coli*

Taidong Bo[1], Chen Wu[1], Zeting Wang[1], Hao Jiang[1], Feiao Wang[1], Ning Chen[1,2,3], Yanjun Li[1,2,3,*]

[1] College of Biotechnology, Tianjin University of Science and Technology, Tianjin, 300457, China

[2] Key Laboratory of Industrial Fermentation Microbiology, Ministry of Education, Tianjin University of Science and Technology, Tianjin, 300457, China

[3] National and Local United Engineering Lab of Metabolic Control Fermentation Technology, Tianjin University of Science and Technology, Tianjin 300457, China

[*] Correspondence: yjli@tust.edu.cn

Figure. S1 The HPLC chromatograph of shikimate and DHS

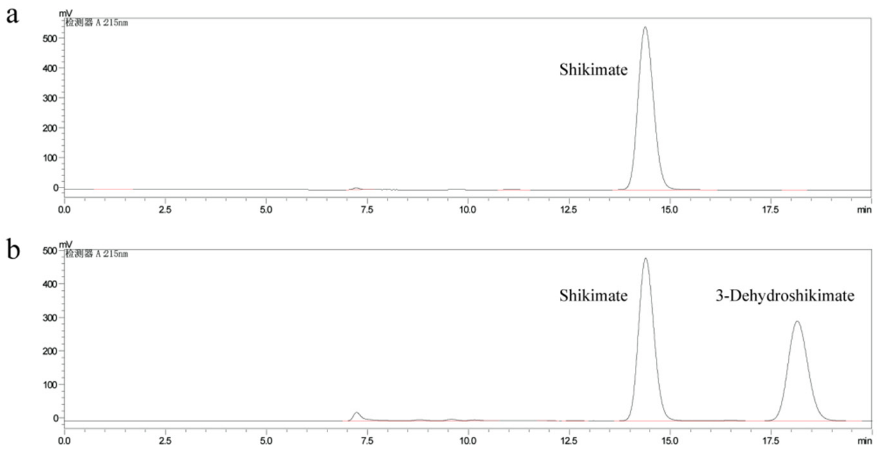

a: standard sample of shikimate, b: fermentation broth containing shikimate and DHS

Table S1. Strains and plasmids used in this study.

Strains/Plasmids	Characteristics	Source
**Strains**		
E. coli DH5α	F$^-$ φ80d*lacZ*ΔM15 Δ(*lacZYA-argF*)U169 *recA1 endA1 hsdR17* (r$_k^-$,m$_k^+$)*deoR supE44* λ$^-$ *thi-1 gyrA*96 *relA1*	Lab stock
dSA00	*E. coli* W3110 Δ*lacI*	Lab stock
dSA01-1	dSA00 *ptsG*::*galP*	This study
dSA01-2	dSA00 *ptsG*::*iolT1* (*Cgl1081*)	This study
dSA02-1	dSA01-1 *rph*::P$_{trc}$-*glk* (*E. coli*)	This study
dSA02-2	dSA01-2 *rph*::P$_{trc}$-*glk* (*C. glutamicum*)	This study
dSA03	dSA02-1 *yeeP*::P$_{trc}$-*tktA*	This study
dSA04	dSA03 *aroL*::P$_{trc}$-*aroB,* Δ*ydiB,* Δ*shiA,* Δ*ydiN*	This study
dSA05-1	dSA04 *tyrR*::P$_{trc}$-*aroG*^{D146N}	This study
dSA05-2	dSA04 *tyrR*::P$_{trc}$-*aroG*^{S180F}	This study
dSA05-3	dSA04 *tyrR*::P$_{trc}$-*aroG*^{S211F}	This study
dSA06-1	dSA05-3 *yghX*::P$_{trc}$-*aroD*	This study
dSA06-2	dSA06-1 *ygaY*::P$_{trc}$-*aroE*	This study
dSA06-3	dSA05-3 *yghX*::P$_{trc}$-*aroD-aroE*	This study
dSA07-1	dSA06-2 Δ*aroK*	This study
dSA07-2	dSA06-2 *aroK*::*aroK*^{M13A}	This study
dSA07-3	dSA06-2 *aroK*::*aroK*^{V471}	This study
dSA07-4	dSA06-2 *aroK*::*aroK*^{F51W}	This study
dSA07-5	dSA06-2 *aroK*::*aroK*^{R60K}	This study
dSA07-6	dSA06-2 *aroK*::*aroK*^{K118A}	This study
dSA07-7	dSA06-2 *aroK*::*aroK*^{R140A}	This study
dSA08	dSA07-2 *ycgH*::P$_{bioFAB104}$-*esaR*^{I70V}	This study
dSA09-1	dSA08 *mbhA*::P$_{L19}$-*esaI*[1]/ pSTV28-*mCherry-egfp*	This study
dSA09-2	dSA08 *mbhA*::P$_{L24}$-*esaI*[1]/ pSTV28-*mCherry-egfp*	This study
dSA09-3	dSA08 *mbhA*::P$_{L31}$-*esaI*[1]/ pSTV28-*mCherry-egfp*	This study
dSA10	dSA09-2 Δ*pykA*, P$_{pykF}$::P$_{esaS}$, P$_{trc}$-*aroG*^{S211F}::P$_{esaR-C}$-*aroG*^{S211F}	This study
**Plasmids**		
pRedCas9	Spe^r, Cas9 and λ Red recombinase expression vector	[15]
pGRB	Amp^r, gRNA expression vector	[15]
pSTV28	Cm^r, p15A ori, medium copy number	Lab stock
pSTV28-*mCherry-egfp*	Cm^r, P$_{esaR-C}$-*mCherry* and P$_{esaS}$-*egfp* expressed in pSTV28	This study

Table S2. Primers used in this study.

Primers	Sequences (5'-3')
ptsG-US	ATCGGTTACTGGTGGAAACTGAC
ptsG-UA	AATTGAGAGTGCTCCTGAGTATGG
ptsG-DS	TAGTGGTTGCTGGTTCTGGTGTTC
ptsG-DA	GCAGAGGATAACGAAGGAAACGC
rph-US	GTTGATTTCCCCTTCGTCACTCT
rph-UA	TGTGTGAAATTGTTATCCGCTCACAATTCCACACATTATACGAGCCGG ATGATTAATTGTCAAAAACAAATGTGGCTGCGCAT
rph-DS	AAAGACTGGGCCTTTCGTTTTATCTGTTGTTTGTCGGTGAACGCTCTC CTGAGTAGGACAAATGGTTTGCGATCTGGAATACGT
rph-DA	TCAGCGTAAACTCGCCAAACT
yeep-US	ACCAAAAAGGGTGAATCTCCG
yeep-UA	TGTGTGAAATTGTTATCCGCTCACAATTCCACACATTATACGAGCCGG ATGATTAATTGTCAATGTCGCCAGAAATACTCATCCAC
yeep-DS	AAAGACTGGGCCTTTCGTTTTATCTGTTGTTTGTCGGTGAACGCTCTC CTGAGTAGGACAAATTTCTGAACCTGTCGTGACTGATG
yeep-DA	GTGGTTCTGTTGTTCCCTGAATG
aroL-US	GTTGCCGATAACGAAATTGTCC
aroL-UA	TGTGTGAAATTGTTATCCGCTCACAATTCCACACATTATACGAGCCGG ATGATTAATTGTCAAAGGTTGTGTCATCGTGGGTTTT
aroL-DS	AAAGACTGGGCCTTTCGTTTTATCTGTTGTTTGTCGGTGAACGCTCTC CTGAGTAGGACAAATCGGGATCGTGGTTTATTTGTGT
aroL-DA	AAATGTGACGCAGATCCCTTGT
ydiB-US	CAATGCCGGAGCCTTTGTTAT
ydiB-UA	CATTCTCAAGGGGTTTCATACCCATTTCGGGCGATAAACTGTGG
ydiB-DS	CCACAGTTTATCGCCCGAAATGGGTATGAAACCCCTTGAGAATG
ydiB-DA	GGTTTTTCTGGCATGGTCTCAC
ydiN-US	TTGCTCTGTTTACCGTCTGCC
ydiN-UA	GCCAAACCGCACATCATTTTCCCCAACAACTGTGGGTAGTTGAGTTC CCTGGGTACATGGCATAA
ydiN-DS	TTATGCCATGTACCCAGGGAACTCAACTACCCACAGTTGTTGGGGAA AATGATGTGCGGTTTGGC
ydiN-DA	AAAGTCCGCTTCACGATAGGC
shiA-US	GCCATAGCATCGCCAATCTGA
shiA-UA	CCGTACTCTGCGGTGATCCAGCCTTATGGCTGATTAGCGGCAAC
shiA-DS	GTTGCCGCTAATCAGCCATAAGGCTGGATCACCGCAGAGTACGGTT GGTTTGCTGGTAGGTGGAT
shiA-DA	CAGGCATCCAGCCAGCAAATA
tyrR-US	AGCCGTTTGCGTCTGTTTAAG
tyrR-UA	GGGCACTTCGGCGTAAAGAT
tyrR-DS	AAAGACTGGGCCTTTCGTTTTATCTGTTGTTTGTCGGTGAACGCTCTC CTGAGTAGGACAAATTGGTGATGTTGCGATCAACGAT
tyrR-DA	TGCACCAGTTCGACCAGATTC

yghX-US	TTGGTAGAGATAATCAGTTCATCGC
yghX-UA	TGTGTGAAATTGTTATCCGCTCACAATTCCACACATTATACGAGCCGG ATGATTAATTGTCAAAGTAATCCAGCAACTCTTGTGGG
yghX-DS	AAAGACTGGGCCTTTCGTTTTATCTGTTGTTTGTCGGTGAACGCTCTC CTGAGTAGGACAAATTAGGTTTATCTCTTACGGGATTACGTC
yghX-DA	AACGAAGGGAAGAAGCATTAGTGTA
aroK-US	CTGGCGATTGATAAGCAGGAG
aroK-UA	CCGTACTCTGCGGTGATCCAGCCTAAATCACCCATCCCTCTGCA
aroK-DS	TGCAGAGGGATGGGTGATTTAGGCTGGATCACCGCAGAGTACGGAT GAACGCAATCCGCTGTATG
aroK-DA	GGACTTGAATGAAACGGACACC
pykA-US	CCCACGATGCGATGAATAAGT
pykA-UA	ACCTTTATCGCGCAGCAGATTAGAATGAAATAACGCCGCGATG
pykA-DS	CATCGCGGCGTTATTTCATTCTAATCTGCTGCGCGATAAAGGT
pykA-DA	CGTTTGATGAAAGTGTGCCGT
ycgH-US	AACAGGCACTAAAGCCCCCAT
ycgH-UA	TAAGTATCCTATAGGTTAGACTTTATGTCGAGTTGTGCCGCTGACGA GTTTA
ycgH-DS	AAAGACTGGGCCTTTCGTTTTATCTGTTGTTTGTCGGTGAACGCTCTC CTGAGTAGGACAAATTGGCTACAATGACACCGCAGT
ycgH-DA	TGGTGCTGTCACTGGCGTTCT
pykF-US	AACTGGACCGCAACTGAAGCA
pykF-UA	GGATACGCTTACACTGTTGTGAGCTGGTGCCGAAGTTGTCAGTGT
pykF-DS	AATTCATTAAAGAGGAGAAAGGATCCATGAAAAAGACCAAAATTGT TTGC
pykF-DA	CCAGGTCACCGTTGTTCAGCA
ygaY-US	CCGATACCAAACTGCCGAATG
ygaY-UA	TGTGTGAAATTGTTATCCGCTCACAATTCCACACATTATACGAGCCGG ATGATTAATTGTCAAGCAATGGCTGGGCGTAATAGT
ygaY-DS	AAAGACTGGGCCTTTCGTTTTATCTGTTGTTTGTCGGTGAACGCTCTC CTGAGTAGGACAAATTACTGGTCTGGTGGCGAGGTT
ygaY-DA	GCAGACGCTGAATAATACGGC
mbhA-US	GCCAGCACGAACATAATCCC
mbhA-UA	TCCACACATTATACGAGCCGATGATTAAGAGGCAACACGGTGGCAGG TTTTGG
mbhA-DS	AAAGACTGGGCCTTTCGTTTTATCTGTTGTTTGTCGGTGAACGCTCTC CTGAGTAGGACAAATGACCAAAAGTGCGTCCGATAC
mbhA-DA	CGGCGTAATCACAAACTGGC
galP-S	GGAGCACTCTCAATTATGCCTGACGCTAAAAAACAGG
galP-A	AACCAGCAACCACTATTAATCGTGAGCGCCTATTTCG
iolT1-S	GGAGCACTCTCAATTATGGCTAGTACCTTCATTCAGGC
iolT1-A	AACCAGCAACCACTATTAGTGCACCTTTCCTTTTCGG
glk_{Eco}-S	CGTATAATGTGTGGAATTGTGAGCGGATAACAATTTCACACAAGGAG ACCAGTTTATGACAAAGTATGCATTAGTCGGTG

glk$_{Eco}$-A	CACCGACAAACAACAGATAAAACGAAAGGCCCAGTCTTTCGACTGA GCCTTTCGTTTTATTTGTTACAGAATGTGACCTAAGGTCTGGC
glk$_{Cgl}$-S	CGTATAATGTGTGGAATTGTGAGCGGATAACAATTTCACACAAGGAG ACCAGTTTATGCCACAAAAACCGGCC
glk$_{Cgl}$-A	CACCGACAAACAACAGATAAAACGAAAGGCCCAGTCTTTCGACTGA GCCTTTCGTTTTATTTGCTAGTTGGCTTCCACTACAGAGCG
tkt-S	CGTATAATGTGTGGAATTGTGAGCGGATAACAATTTCACACAAGGAG ACCAGTTTATGTCCTCACGTAAAGAGCTTGC
tkt-A	CACCGACAAACAACAGATAAAACGAAAGGCCCAGTCTTTCGACTGA GCCTTTCGTTTTATTTGTTACAGCAGTTCTTTTGCTTTCGC
aroB-S	CGTATAATGTGTGGAATTGTGAGCGGATAACAATTTCACACAAGGAG ACCAGTTTATGGAGAGGATTGTCGTTACTCTCG
aroB-A	CACCGACAAACAACAGATAAAACGAAAGGCCCAGTCTTTCGACTGA GCCTTTCGTTTTATTTGTTACGCTGATTGACAATCGGC
aroD-S	CGTATAATGTGTGGAATTGTGAGCGGATAACAATTTCACACAAGGA GACCAGTTTATGAAAACCGTAACTGTAAAAGATCTCG
aroD-A	CACCGACAAACAACAGATAAAACGAAAGGCCCAGTCTTTCGACTGA GCCTTTCGTTTTATTTGTTATGCCTGGTGTAAAATAGTTAATAC
aroE-S	CGTATAATGTGTGGAATTGTGAGCGGATAACAATTTCACACAAGGAG ACCAGTTTATGGAAACCTATGCTGTTTTTGG
aroE-A	CACCGACAAACAACAGATAAAACGAAAGGCCCAGTCTTTCGACTGA GCCTTTCGTTTTATTTGTCACGCGGACAATTCCTCC
aroG-S	CGTATAATGTGTGGAATTGTGAGCGGATAACAATTTCACACAAGGA GACCAGTTTATGAATTATCAGAACGACGATTTACG
aroG-1 (D146N)	TGGGGTGATCATATTGAGAAACTCACCTGC
aroG-2 (D146N)	GCAGGTGAGTTTCTCAATATGATCACCCCA
aroG-3 (S180F)	GAAGCCGACCGGACAAAAAAGCCCTGATGC
aroG-4 (S180F)	GCATCAGGGCTTTTTTGTCCGGTCGGCTTC
aroG-5 (S211F)	TTACGAACAGGAAGCAGTGCG
aroG-6 (S211F)	CGCACTGCTTCCTGTTCGTAA
aroG-A	CACCGACAAACAACAGATAAAACGAAAGGCCCAGTCTTTCGACTGA GCCTTTCGTTTTATTTGTTACCCGCGACGCGCTTT
aroK-1 (M13A)	GCTTTTTCCGGCACCTGCAGGCCCAACCAGAAA
aroK-2 (M13A)	TTTCTGGTTGGGCCTGCAGGTGCCGGAAAAAGC
aroK-3 (V47I)	ATCGAAAACCCAGCCAATATCAGCTCCGGTTCG
aroK-4 (V47I)	CGAACCGGAGCTGATATTGGCTGGGTTTTCGAT
aroK-5 (F51W)	TTCGCCTTCTAAATCCCAAACCCAGCCCACATC
aroK-6 (F51W)	GATGTGGGCTGGGTTTGGGATTTAGAAGGCGAA
aroK-7 (R60K)	CTTTTCTTCGCGATCTTTGAAGCCTTCTTCGCC
aroK-8 (R60K)	GGCGAAGAAGGCTTCAAAGATCGCGAAGAAAAG
aroK-9 (K118A)	CAGCAACGGGCGTTTTGCATCACGCTGCGTGCG
aroK-10 (K118A)	CGCACGCAGCGTGATGCAAAACGCCCGTTGCTG
aroK-11 (R140A)	TTCATACAGCGGATTTGCTTCATTGGCCAACG
aroK-12 (R140A)	CGTTGGCCAATGAAGCAAATCCGCTGTATGAA

esaR-US	TCGACATAAAGTCTAACCTATAGGATACTTACAGCCATCAATTCATT<br>AAAGAGGAGAAAGGATCCATGTTTTCTTTTTTCCTTGAAAAT
esaR-UD (I70V)	TAAAGGCCGTGAGAACAACCGGATCGGTCAG
esaR-DS (I70V)	CTGACCGATCCGGTTGTTCTCACGGCCTTTA
esaR-DA	CACCGACAAACAACAGATAAAACGAAAGGCCCAGTCTTTCGACTGA<br>GCCTTTCGTTTTATTTGTTACCTGGCCGCTGACGCTG
esaI-S	ATGCTTGAACTGTTTGACGTCAGT
esaI-A	CACCGACAAACAACAGATAAAACGAAAGGCCCAGTCTTTCGACTGA<br>GCCTTTCGTTTTATTTGTTAGACCGGCAGCGTCAGC
pGRB-S	TTTTAGAGCTAGAAATAGCAAGTTAA
pGRB-A	ATTATACCTAGGACTGAGC
pSTV28-S	AAAGACTGGGCCTTTCGTTTTATCTGTTGTTTGTCGGTGAACGCTCTC<br>CTGAGTAGGACAAATCAACTTATATCGTATGGGGCTGAC
pSTV28-A	CTATCGTCGCCGCACTTATGA
P_{esaS}-S	TCATAAGTGCGGCGACGATAGGCAGATTGAGTAACCGTGAATGTT
P_{esaS}-A	CCTCGCCCTTGCTCACCATGGATCCTTTCTCCTCTTTAATGAATT
$P_{esaR\text{-}C}$-S	AATTCATTAAAGAGGAGAAATGGTGAGCAAGGGCGAGG
$P_{esaR\text{-}C}$-A	GGATACGCTTACACTGTTGTGAGCTTACTTGTACAGCTCGTCCATGCC
mCherry-S	GGCATGGACGAGCTGTACAAGTAAGCTCACAACAGTGTAAGCGTAT<br>CC
mCherry-A	CCTCGCCCTTGCTCACCATGGATCCTTTCTCCTCTTTAATGAATT
egfp-S	AATTCATTAAAGAGGAGAAAGGATCCATGGTGAGCAAGGGCGAGG
egfp-A	CACCGACAAACAACAGATAAAACGAAAGGCCCAGTCTTTCGACTGA<br>GCCTTTCGTTTTATTTGTTACTTGTACAGCTCGTCCATGCC

Table S3. The gRNA targeting sequences used in this study.

gRNA	Sequences (5'-3')
pGRB-*ptsG*	TCTAAACACCTGGCGGATAC
pGRB-*rph*	GGCTGGATCACCGCAGAGTA
pGRB-*yeep*	ACAGAATATTCGCGAAAAAA
pGRB-*aroL*	TTGCCGATTCGCTTAACCGT
pGRB-*ydiB*	GCCCTCAAAATGCGCGGAAC
pGRB-*ydiN*	TCTCCGATAAGTTTGGTCGT
pGRB-*shiA*	AATAGGTGCCCACCACCCAA
pGRB-*tyrR*	CTCGATCTACTCGTGCTAAG
pGRB-*yghX*	CCCACTTTGCCTGTCGCTTG
pGRB-*aroK*	GCTTCCAGAACTTCACGCGG
pGRB-*pykA*	ATCTGTTGCTGGGCCTAACG
pGRB-*ycgH*	ATGCGTCTGAACGACCGTGC
pGRB-*pykF*	CACCACCACTTTCGTAATAC
pGRB-*ygaY*	CTCAACTACCCACAGTTGTT
pGRB-*mbhA*	TACCGGGCATACCGATGCGA

Table S4. Gene and promoter sequences involved in the QS circuits.

Genes/ Promoters	Sequence (5'-3')
esaR	TCGACATAAAGTCTAACCTATAGGATACTTACAGCCATCAATTCATTAAAGAG GAGAAAGGATCCATGTTTTCTTTTTTCCTTGAAAATCAAACAATAACGGATACG CTTCAGACTTACATACAGAGAAAGTTATCTCCGCTGGGTAGTCCGGATTACGCTT ACACTGTTGTGAGCAAAAAAAATCCTTCAAATGTTCTGATTATTTCCAGTTATCC TGACGAATGGATTAGGTTATACCGCGCTAACAACTTTCAGCTGACCGATCCGGTT ATTCTCACGGCCTTTAAACGCACCTCGCCGTTTGCCTGGGATGAGAATATTACGC TGATGTCCGACCTGCGGTTCACCAAAATTTTCTCTTTATCCAAGCAATACAACAT CGTTAACGGCTTTACCTATGTCCTGCATGACCACATGAACAACCTTGCTCTGTTG TCCGTGATCATTAAAGGCAACGATCAGACTGCGCTGGAGCAACGCCTTGCTGCC GAACAGGGCACGATGCAGATGCTGCTGATTGATTTAACGAGCAGATGTACCGC CTGGCCGGTACCGAAGGCGAGCGAGCCCCGGCGTTAAATCAGAGCGCGGACAA AACGATATTTCCTCGCGTGAAAATGAGGTGTTGTACTGGGCGAGTATGGGCAA AACCTATGCTGAGATTGCCGCTATTACGGGCATTTCTGTGAGTACCGTGAAGTTT CACATCAAGAATGTGGTCGTGAAACTGGGCGTCAGTAACGCCCGACAGGCTAT CAGACTGGGTGTAGAACTGGATCTTATCAGACCGGCAGCGTCAGCGGCCAGGT AGTGA
esaI	ATGCTTGAACTGTTTGACGTCAGTTACGAAGAACTGCAAACCACCCGTTCAGA AGAACTTTATAAACTTCGCAAGAAAACATTTAGCGATCGTCTGGGATGGGAAG TCATTTGCAGTCAGGGAATGGAGTCCGATGAATTTGATGGGCCCGGTACACGT TATATTCTGGGAATCTGCGAAGGACAATTAGTGTGCAGCGTACGTTTTACCAG CCTCGATCGTCCCAACATGATCACGCACACTTTTCAGCACTGCTTCAGTGATGT CACCCTGCCCGCCTATGGTACCGAATCCAGCCGTTTTTTTGTCGACAAAGCCCG CGCACGTGCGCTGTTAGGTGAGCACTACCCTATCAGCCAGGTCCTGTTTTTAGC GATGGTGAACTGGGCGCAAAATAATGCCTACGGCAATATCTATACGATTGTCA GCCGCGCGATGTTGAAAATTCTCACTCGCTCTGGCTGGCAAATCAAAGTCATT AAAGAGGCTTTCCTGACCGAAAAGGAACGTATCTATTTGCTGACGCTGCCAGC AGGTCAGGATGACAAGCAGCAACTCGGTGGTGATGTGGTGTCACGTACGGGCT GTCCGCCCGTCGCAGTCACTACCTGGCCGCTGACGCTGCCGGTCTGATAA
P_{esaS} [52]	GCTCACAACAGTGTAAGCGTATCCGTTATTGTTTGATTTTCAAGGAAAAAAGAA AACATTCAGGCTCCATGCTGCTTCTTTTACTTAACGTGGACTTAACCTGCACTAT AGTACAGGCAAGATGATACTTAAGAGTAACTTACAATGAATCATTCAGAGGTTA CAATGGCTTCAGTTGTTTAGCCAATTCATTAAAGAGGAGAAAGGATCC
$P_{esaR\text{-}C}$ [59]	GCAGATTGAGTAACCGTGAATGTTTGTACAAATGTTTCAAAGATGTTACTATGAG TGTCCCGGCCAGCATCACTTTATATTTGTGACGTCTGGCCGGACGTTTTCCCTA GTGTTGGCTGTTTTAGCGACCTGGCCGTACAGGTCAGGTTTTTTTTTACCGCTAA ACAACTGAAGCCATTGTAACCTCTGAATGATTCATTGTAAGCCTGTACTATAGTG CAGGTTGCCTGTACTATAGTGCAGGTAAGTCCACGTTAAGTAAAAGAAGCAGCC AATTCATTAAAGAGGAGAAAGGATCC
P_{L19} [52]	TTCACTTTTAATCATCCGGCTCGTATAATGTGTGGAGGGCCCAAGTTCACTTAAA AAGGAGATCAACAATGAAAGCAATTTTCGTACTGAAACATCTTAATCATGCCTA GGAAGTTTTCTA
P_{L24} [52]	TTGCCTCTTAATCATCGGCTCGTATAATGTGTGGAGGGCCCAAGTTCACTTAAAA AGGAGATCAACAATGAAAGCAATTTTCGTACTGAAACATCTTAATCATGCGAGG GATGGTTTCTA
P_{L31} [52]	TTGACATCAGGAAAATTTTTCTGTATAATGTGTGGAGGGCCCAAGTTCACTTAA AAAGGAGATCAACAATGAAAGCAATTTTCGTACTGAAACATCTTAATCATGCCT AGGAAGTTTTCTA
